# Polylysine-Containing Hydrogel Formulation of Fuzapladib, Inhibitor of Leukocyte-Function Associated Antigen-1 (LFA-1) Activation, for Sustained Release

**DOI:** 10.3390/molecules28145325

**Published:** 2023-07-11

**Authors:** Koji Higuchi, Kohei Yamada, Tsubasa Kihara, Keisuke Makino, Kenta Sasaki, Takeshi Shindo, Hiroshi Shikama, Hideyuki Sato, Satomi Onoue

**Affiliations:** 1 Laboratory of Biopharmacy, School of Pharmaceutical Sciences, University of Shizuoka, 52-1, Yada, Suruga-ku, Shizuoka-shi 422-8526, Shizuoka, Japan; 2 Healthcare Business Headquarters, Ishihara Sangyo Kaisha, Ltd., 2-3-1, Nishishibukawa, Kusatsu-shi 525-0025, Shiga, Japan

**Keywords:** acute pancreatitis, fuzapladib, hydrogel, polylysine, sustained release

## Abstract

The aim of the present study was to develop an injectable hydrogel (HG) formulation of fuzapladib sodium (FZP), an animal drug for acute pancreatitis (AP), with the use of polyethyleneoxide (PEO) and polylysine (pLys), a cationic polymer. A mixture of pLys and FZP was added to PEO to prepare an HG formulation, and the formulation was optimized by release test and viscosity measurements. Circular dichroism (CD) and infrared absorption (IR) spectral analyses were applied to clarify the intermolecular interactions between FZP and pLys. The pharmacokinetic behavior of FZP was evaluated after a subcutaneous administration of FZP samples (2.0 mg-FZP/kg) to rats. Although the immediate release of FZP was observed for the HG formulation, the addition of pLys at a 20-fold amount of FZP or higher led to the sustained release of FZP. Considering release behavior, the concentration of pLys was optimized as 100-fold that of FZP in the HG formulation. CD and IR spectroscopic analyses of FZP and/or pLys demonstrated an intermolecular interaction between FZP and pLys, as evidenced by the slight spectral transition. After a subcutaneous administration of HG formulation containing pLys to rats, compared with FZP alone, significant differences were observed in the pharmacokinetic behavior with a decrease of *C*_max_ from 2.3 to 0.9 mg/mL and slower elimination kinetics. HG formulation using pLys might be a viable dosage option for FZP for the treatment of AP in animals.

## 1. Introduction

Fuzapladib sodium (FZP, development code: IKV-741) is an active pharmaceutical ingredient of a therapeutic agent for canine acute pancreatitis (AP) approved in Japan [1] and the US [2], and is available as a lyophilized formulation for intravenous injection. Its mode of action is to suppress the activation of one of integrin, leukocyte-function-associated antigen type 1 (LFA-1), a cell adhesion molecule on neutrophils, and thereby FZP can inhibit the migration of neutrophils into inflammatory sites, leading to potent prevention of inflammatory symptoms [3]. According to clinical reports, the main symptoms of AP in companion animals are weakness, diarrhea, polyuria/polydipsia, anorexia, and most notably, vomiting [4,5,6]; therefore, oral medication would be inappropriate for the clinical treatment of AP. as Along with dogs, cats suffer from AP [7,8,9], and so the indication for FZP should be expanded to cats. In our previous study, the metabolism of FZP in cats was found to be much faster than that in dogs, suggesting species differences in pharmacokinetic behavior [10]. Upon injection of FZP into cats, it might be challenging to maintain the blood concentration of FZP required to achieve clinical outcomes. To overcome this problem, a new injectable formulation of FZP should be developed to offer long-lasting systemic exposure and has better therapeutic potential for the treatment of AP in cats. 

A number of efforts have been made to establish novel injectable formulations with long-lasting systemic exposure, including liposome formulations [11], polymer formulations [12], micellar formulations [13], hydrogels (HG) [14,15], and organogels [16]. Among them, HG might be superior in terms of manufacturing costs. The HG formulation can be defined as networks of highly water-absorbent natural or synthetic polymers [15], and it can control drug release through the encapsulation effect of its three-dimensional structure. Herein, an HG formulation approach might be an effective dosage option for FZP with long-lasting systemic exposure. However, FZP is a very small molecule, so controlled release via HG is expected to be challenging since molecular FZP can pass through the gel matrix with ease. When inner drugs are ionic molecules, another potential advantage of HG is the improvement of sustained-release properties by the addition of oppositely charged polymers for the formation of a complex with inner ionic molecules. In theory, FZP, an anionic molecule, would form a large complex with cationic polymers, and then can be captured in the gel matrix. Polylysine (pLys), a cationic polymer, has been employed as a functional excipient for food [17] and pharmaceuticals [18], and it might exhibit weak intramolecular interactions with FZP, forming a large complex. The combined use of the HG strategy and pLys-based complex might provide a controlled release, injectable formulation of FZP; however, its feasibility remains unclear. 

In the present investigation, a polyethyleneoxide (PEO)-based HG formulation of FZP (HG/FZP) was developed, in which pLys was employed as a cationic matrix for intermolecular interactions to confer controlled release properties. The amount of pLys was optimized with a focus on sustained release behavior. Possible intermolecular interactions between FZP and pLys in the formulation were assessed by circular dichroic (CD) and infrared absorption (IR) spectra. After subcutaneous administration of FZP or the new HG formulation to rats, the pharmacokinetic behavior of FZP was also characterized.

## 2. Results and Discussion

### 2.1. Delayed Dissolution Behavior of HG/FZP with pLys (HG/FZP-pLys)

**Figure 1:**
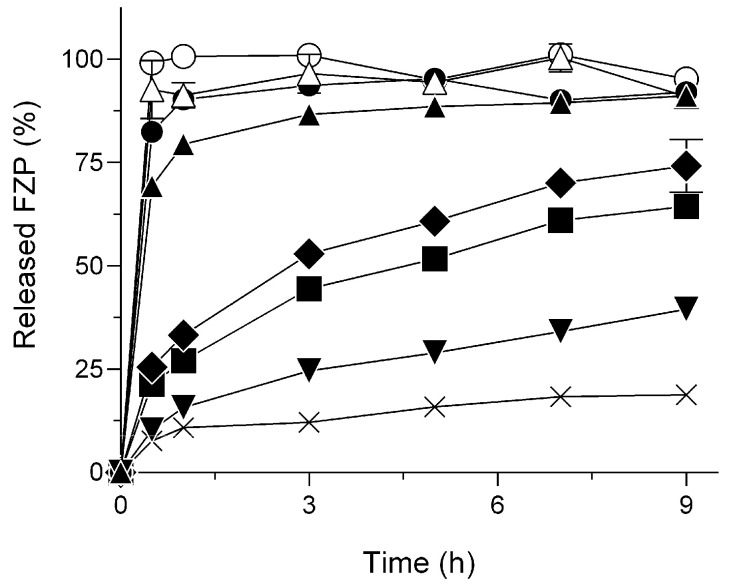
Release behavior of FZP from HG formulations (pH7.4, 37 °C). ○, FZP; △, HG/FZP; ●, HG/FZP-pLys5; ▲, HG/FZP-pLys10; ◆, HG/FZP-pLys20; ■, HG/FZP-pLys50; ▼, HG/FZP-pLys100; and ×, HG/FZP-pLys150. Data represent mean ± S.D. (*n* = 3).

HG/FZP and HG/FZP-pLys samples at different ratios of pLys to FZP were prepared with the use of PEO (Table 1), and they showed high viscosity and no precipitations. To clarify the role of pLys in the sustained release performance of FZP, comparative dissolution studies were performed (Figure 1). HG formulations without pLys (HG/FZP) have an insufficient capacity to retain FZP, and there are no significant differences in dissolution behavior between HG/FZP and HG/FZP with low concentrations of pLys (HG/FZP-pLys5 and HG/FZP-pLys10). However, HG/FZP containing pLys at an amount that is 20-fold or higher exhibited sustained release behavior, and the dissolution rate of HG/FZP-pLys100 at 9 h was reduced to ca. 40%. Interestingly, the dissolution rate of HG/FZP-pLys150 reached a plateau at ca. 15% after 3 h, which may result in an incomplete release after injection. Thus, HG/FZP-pLys100 with continuous and delayed dissolution behavior was selected as a favorable formulation in order to provide long-lasting systemic exposure and thereby a longer duration of the pharmacological action of FZP.

**Figure 2:**
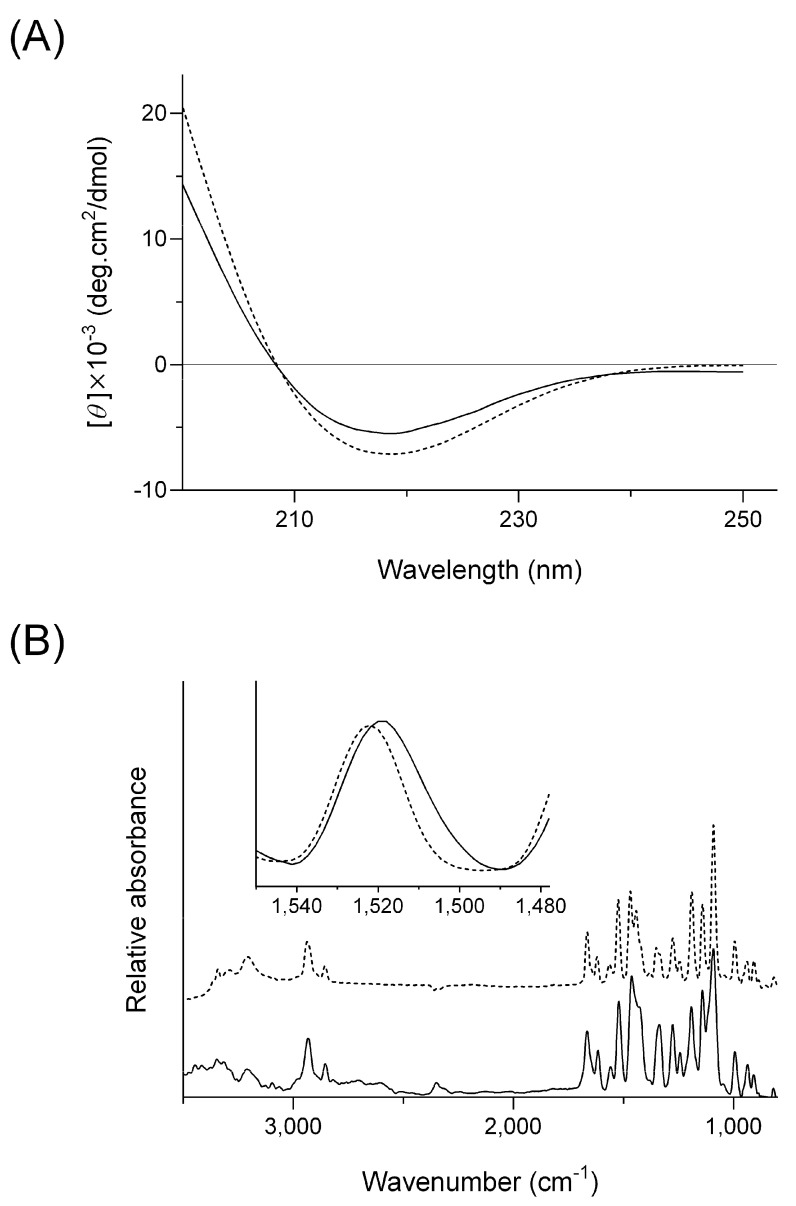
CD and IR spectral analyses of FZP and/or pLys. (**A**) CD spectra of pLys with or without FZP. Solid line, pLys with FZP; and dashed line, pLys without FZP. (**B**) Baseline-corrected and normalized IR spectra of FZP in KBr pellet with or without pLys after Kubelka-Munk conversion. Solid line, the differential spectrum by subtracting the pLys spectrum from the mixture of FZP and pLys; and dashed line, FZP alone.

In the present study, the viscosities of HG/FZP and HG/FZP-pLys samples were measured because the viscosity of HG formulations could theoretically affect the release behavior of inner drugs. However, the viscosities of HG/FZP and HG/FZP-pLys samples were found to be ranging from ca. 100 to 127 mPa.s, suggesting that viscosity would have no major impact on the sustained release behavior of HG/FZP-pLys samples. According to the sustained release behavior of anionic FZP in the presence of cationic pLys, there might be complex formations in the HG formulations; therefore, CD and IR spectra were measured for a clarification of possible intramolecular interactions (Figure 2).

In theory, CD is a measurement of the difference in absorption between left and right-turning circular polarizations, and in CD analysis on proteins, the observed optical rotation might be attributable to the secondary structure of the peptide chain [19]. Herein, CD spectral analysis was identified as an effective means for investigating the denaturation state of proteins [20]. The CD spectral pattern on the mixture of FZP and pLys was measured and compared with that of pLys (Figure 2A). There appeared to be a slight spectral transition of pLys in the presence of FZP, and a-helical and b-strand moieties were calculated to be 6.2 and 30.6% for pLys alone and 3.2 and 36.5% for pLys with FZP, respectively. The slight change in the secondary structure of pLys would be indicative of a possible intermolecular interaction between FZP and pLys. 

For further characterization, IR spectral analyses were also employed to investigate the possible interaction. In the IR spectral analysis, the characteristic absorption bands of compounds can be shifted upon intermolecular interactions [21]. The IR spectral pattern on the mixture of FZP and pLys in the KBr pellet was measured and compared with that of pLys. To confirm the IR spectral transition of FZP in the presence of pLys, the differential spectrum was obtained by subtracting the pLys spectrum from the mixture of FZP and pLys (Figure 2B). The IR absorption band at ca. 1520 cm^−1^ can be derived from the pyridine ring of FZP, and the band, in the presence of pLys, was found to have shifted to the lower energy side by ca. 5 cm^−1^. The sulfonamide group of FZP, neighboring the pyridine ring, can be responsible for electrostatic interaction with pLys; therefore, there might be spectral interference on the pyridine ring due to the intermolecular interactions between FZP and pLys.

On the basis of the transition in the CD and IR spectral patterns, FZP and pLys in the HG formulation would form a complex through intermolecular interaction, possibly leading to a delay in the release behavior. 

### 2.2. Pharmacokinetic Behavior of HG/FZP-pLys100 in Rats

**Figure 3:**
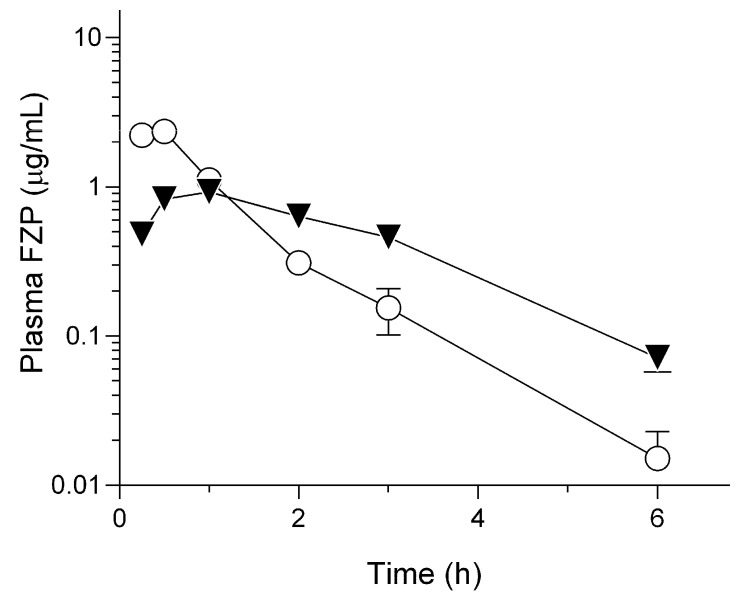
Pharmacokinetic behavior of FZP after subcutaneous administration of FZP and HG/FZP-pLys100 (2.0 mg-FZP/kg) in rats. Data represent mean ± S.E. (*n* = 4).

The sustained release behavior of HG/FZP-pLys100, taken together with spectral data, prompted us to examine pharmacokinetic characterization to assess in vivo controlled release properties. A pharmacokinetic study on HG/FZP-pLys100 in rats was performed and compared with that of FZP (Figure 3 and Table 2). The dosage of 2.0 mg-FZP/kg used in this study was equivalent to a five-fold dosage of the commercially available injection product [2], and the dose for animal testing was defined based on a careful consideration of LC analytical sensitivity. After subcutaneous injection, FZP could be absorbed immediately and eliminated with t_1/2_ of 0.7 ± 0.2 h after showing a t_max_ value of 0.4 ± 0.1 h; however, HG/FZP-pLys100 exhibited an evident absorption phase with a *T*_max_ value of 0.9 ± 0.2 h, followed by slow elimination with t_1/2_ of 1.7 ± 0.4 h. *C*_max_ values for FZP and HG/FZP-pLys100 were calculated to be 2.3 ± 0.4 and 0.9 ± 0.1 mg/mL, respectively. The *C*_max_ value decreased by more than half and the t_1/2_ value increased more than twice, indicating that HG/FZP-pLys100 had a significant sustained release effect. The absolute bioavailability of both samples showed almost the same value of ca. 90%, suggesting that FZP might not be captured within HG.

Previous investigations demonstrated evident species differences in the pharmacokinetic behavior of FZP after subcutaneous injection; in particular, rapid elimination and short-term systemic exposure of FZP were observed in cats, possibly leading to limited clinical outcomes [10]. Fundamental knowledge from the present study might offer hope for extending the blood retention time of FZP in cats; however, much longer blood retention times for FZP might be required for efficient clinical treatment of feline AP. Further formulation studies are needed to achieve a much longer systemic exposure of FZP. For example, drug-releasing behavior from HG might be affected by the structure and size of polymers and their viscosity. As alternatives to pLys, other cationic polymers could be available for a stronger electrostatic interaction with FZP, and polyarginine would be one candidate because of its higher p*K*_a_ value compared with pLys. With further optimization, the HG formulation may be a promising dosage form for FZP to provide a reliable clinical treatment of feline AP.

## 3. Materials and Methods

### 3.1. Materials

FZP sodium hydrate was synthesized as reported previously [22]. PEO with an average molecular weight of 8000 kDa was purchased from Sigma-Aldrich Japan (Tokyo, Japan). The e-pLys was supplied by Okuno Industry Co., Ltd. (Osaka, Japan). All other chemicals and solvents (liquid chromatography grade) were purchased from commercially available sources.

### 3.2. Preparation

HG/FZP and HG/FZP-pLys samples were prepared in a beaker. The 0.5 mL of water containing 20 mg PEO and the same volume of water containing 2 mg of FZP and pLys at different ratios to FZP were mixed by agitation using a magnetic stirrer (Table 1). HG/FZP without pLys was also prepared as a control HG formulation for comparison.

### 3.3. Release Test

Release test of HG/FZP samples was performed in 30 mL of phosphate-buffered saline (pH 7.4) at 37 °C with horizontal shaking of 30 strokes/min. Three hundred mL of aliquots were taken at determined periods (0.5, 1, 3, 5, 7, and 9 h) and filtered with a 0.2 mm filter. The filtrates were diluted with methanol, and the FZP concentration in samples was measured using a high-performance liquid chromatography (HPLC) system equipped with a photodiode array detector with a fixed wavelength of 276 nm. The HPLC system consisted of an SPD-M10A diode array detector, LC-20AD pump, SIL-20AC autosampler, and a CTO-20A column oven controlled by LC Solution software ver. 5.101 (Shimadzu Corp., Kyoto, Japan). FZP in analytes was separated with a CAPCELL PAK SG120Å column (particle size: 5.0 μm, column size: 4.6 mm I.D. × 120 mm; Shiseido, Tokyo, Japan). The isocratic mobile phases were 0.1% formic acid and methanol, and their ratio was set at 40:60 with a flow rate of 1 mL/min. The retention time of FZP was 4.5 min.

### 3.4. Viscosity Measurement

The viscosity of HG/FZP and HG/FZP-pLys samples was assessed by SV-10 (A&D, Tokyo, Japan). Each HG/FZP sample was put into a 45 mL polycarbonate sample cup, and the temperature of HG was equilibrated to room temperature.

### 3.5. Circular Dichroism Spectroscopy

CD spectra of pLys were obtained using a J-820 circular dichroism spectropolarimeter (JASCO, Tokyo, Japan) in the presence or absence of FZP. The pLys was dissolved at 1 mM in water with or without FZP at 1 mM. The sample solutions were put into quartz cuvettes with an optical pass length of 0.1 cm. The CD spectra were recorded in triplicate from 190 to 250 nm with a 1.0 nm spectral bandwidth at room temperature. The secondary structure of pLys in each sample was determined using K2D3 online software [23].

### 3.6. Fourier Transformation Infrared Spectroscopy

IR spectra were obtained for pLys, FZP, and their freeze-dried mixture by the KBr method using IRPrestige-21 (Shimadzu Corp. Kyoto, Japan). Each sample was mixed with 100 mg of KBr powder, and they were manually pressed to prepare pellets. The wavenumber range for analysis was set to 4000 to 500 cm^−1^ at intervals of 1 cm^−1^. 

### 3.7. Animals

Male Sprague–Dawley rats (210 ± 20 g, 7 weeks of age) were purchased from Japan SLC (Shizuoka, Japan) and housed with free access to food and water in the laboratory at a controlled temperature (24 ± 1 °C), humidity (55 ± 5%), and light/dark cycle (12/12 h). All procedures used in the present study were approved by the Institutional Animal Care and Ethical Committee of the University of Shizuoka.

### 3.8. Pharmacokinetic Study on Subcutaneous FZP Samples

Each FZP sample, including FZP aqueous solution and HG/FZP-pLys, was subcutaneously administered to rats at 2.0 mg-FZP/kg. The blood samples were collected from a tail vein at determined periods (0.25, 0.5, 1, 2, 3, and 6 h), followed by centrifugation at 10,000× *g* for 10 min at 4 °C. The plasma samples were stored below −20 °C until analysis. The FZP concentration in plasma samples was determined with the use of a Waters Acquity ultra-performance liquid chromatography (UPLC) system (Waters, Milford, MA, USA). The UPLC system consisted of a binary solvent manager, sample manager, column compartment, and single quadrupole detector (SQD) connected with MassLynx software ver. 4.2. FZP in analytes was separated from impurities in plasma samples using an Acquity UPLC BEH C18 column (particle size: 1.7 mm, column size: 2.1 × 50 mm; Waters). The column temperature and flow rate were set at 40 °C and 0.25 mL/min, respectively. The mobile phases for gradient elution were 0.1% formic acid (A) and methanol (B), and the gradient conditions were 0–0.5 min, 60% B; 0.5–3.5 min, 60–80% B; 3.5–4.5 min, 95% B; and 4.5–5.5 min, 60% B. Prior to analysis, 50 mL of plasma samples were mixed with 150 mL of methanol-containing tranilast as an internal standard for deproteinization. The supernatants obtained after centrifugation were filtered with a 0.2 mm filter to prepare samples for UPLC analysis. Pharmacokinetic parameters were calculated using GraphPad Prism software ver. 10.0.0 for Windows (GraphPad Software, Boston, MA, USA).

### 3.9. Statistical Analysis

For statistical comparison, data were subjected to one-way analysis of variance (ANOVA) with pairwise comparison by Fisher’s least significant difference procedure. A p-value of less than 0.05 was considered significant for all analyses.

## 4. Conclusions

In the present study, an HG formulation of FZP was newly developed to control the pharmacokinetic behavior of FZP after subcutaneous injection. The addition of pLys into HG resulted in a significant delay in the release behavior of FZP, and results from spectroscopic analyses were indicative of intermolecular interactions between FZP and FZP. Compared with FZP alone, there was a longer systemic exposure of FZP after the subcutaneous administration of the newly prepared HG formulation. Based on these findings, the combined use of the HG formulation approach and cationic polymer might be efficacious for pharmacokinetic control of FZP in the clinical treatment of animal AP.

## Figures and Tables

**Table 1:** Compositions of FZP-loaded HGs.

	Composition			
	PEO (mg)	FZP (mg)	pLys (mg)	Water (mL)
HG/FZP	10	1	0	1
HG/FZP-pLys5	10	1	5	1
HG/FZP-pLys10	10	1	10	1
HG/FZP-pLys20	10	1	20	1
HG/FZP-pLys50	10	1	50	1
HG/FZP-pLys100	10	1	100	1
HG/FZP-pLys150	10	1	150	1

**Table 2:** Pharmacokinetic parameters of FZP samples (2.0 mg-FZP/kg) in rats.

	*C*_max_ (mg/mL)	t_max_ (h)	t_1/2_ (h)	MRT (h)	AUC_0–inf_ (mg·h/mL)	BA (%)
FZP	2.3 ± 0.4	0.4 ± 0.1	0.7 ± 0.2	1.1 ± 0.2	2.9 ± 0.2	90.2
HG/FZP-pLys100	0.9 ± 0.1	0.9 ± 0.2	1.7 ± 0.4	2.1 ± 0.1	2.9 ± 0.4	88.0

*C*_max_: maximum concentration; t_max_: time to maximum concentration; t_1/2_: half-life; MRT, mean residence time; AUC_0–inf_: area under the curve of blood concentration vs. time from t = 0 to t = ∞ after administration; and BA: oral bioavailability. Values are expressed as mean ± S.E. (*n* = 4).

## Data Availability

Not applicable.
